# A global-scale data set of mining areas

**DOI:** 10.1038/s41597-020-00624-w

**Published:** 2020-09-08

**Authors:** Victor Maus, Stefan Giljum, Jakob Gutschlhofer, Dieison M. da Silva, Michael Probst, Sidnei L. B. Gass, Sebastian Luckeneder, Mirko Lieber, Ian McCallum

**Affiliations:** 1 Institute for Ecological Economics, Vienna University of Economics and Business (WU), Vienna, Austria; 2 Ecosystems Services and Management, International Institute for Applied Systems Analysis (IIASA), Laxenburg, Austria; 3 Federal University of Pampa (UNIPAMPA), Itaqui, Brazil

**Keywords:** Sustainability, Environmental economics, Environmental impact

## Abstract

The area used for mineral extraction is a key indicator for understanding and mitigating the environmental impacts caused by the extractive sector. To date, worldwide data products on mineral extraction do not report the area used by mining activities. In this paper, we contribute to filling this gap by presenting a new data set of mining extents derived by visual interpretation of satellite images. We delineated mining areas within a 10 *km* buffer from the approximate geographical coordinates of more than six thousand active mining sites across the globe. The result is a global-scale data set consisting of 21,060 polygons that add up to 57,277 *km*^2^. The polygons cover all mining above-ground features that could be identified from the satellite images, including open cuts, tailings dams, waste rock dumps, water ponds, and processing infrastructure. The data set is available for download from 10.1594/PANGAEA.910894 and visualization at www.fineprint.global/viewer.

## Abstract (summary)

**Table:** 

Measurement(s)	terrestrial mining
Technology Type(s)	satellite imaging
Sample Characteristic - Environment	land
Sample Characteristic - Location	Earth (planet)

Machine-accessible metadata file describing the reported data: 10.6084/m9.figshare.12594248

## Background & Summary

Global extraction of minerals grew at an unprecedented pace in the past decades, causing a wide range of social and environmental impacts around the world^1–3^. Growing demand for essential minerals and declining quality of ores^4–6^ lead to larger volumes of unused material extracted and disposed^7^, increasing appropriation of land^8,9^. The direct land used by mining is a crucial indicator of environmental pressure, which is closely associated with a range of negative impacts, including fragmentation and degradation of ecosystems and biodiversity loss^10–14^. Such an indicator supports the implementation and monitoring of several Sustainable Development Goals (SDGs), as mining impacts on biodiversity and ecosystem services can be reduced by limiting mining areas^15^. Data on land use of mining is also important to further develop land footprint indicators that inform about land required along global supply chains to satisfy final consumption of products^16,17^. Yet, to date information about mining areas worldwide is not available.

Databases on the global mining sector are regularly updated by national geological services, mining industries, associations, and information services^18,19^. These databases, however, focus on commodities production, not on land use or other environmental aspects. They include, for example, commodity classifications, produced volumes, and approximate location of the sites, but not their geographic extents. These data sources alone are therefore not sufficient for a comprehensive assessment of the impacts related to the direct land use of global mining.

Satellite images are an important source of information on mining extents complementing surveys and statistics. Visual interpretation of satellite images^9^, for example, has been applied to map the 295 most relevant mining sites in terms of commodities production across the world^20,21^. This approach is effective and precise but can be costly and time-intensive, therefore, posing challenges to producing comprehensive accounts of global mining areas. Alternatively, automated classification algorithms to monitor land-use changes have rapidly advanced due to the increasing availability of satellite images and computational infrastructure^22–26^. These developments have helped to map mining extents in many regions^27–31^. However, scaling automated classification is difficult, as current state-of-the-art algorithms require a large amount of labeled examples^32^, which are usually not available.

In this work, we contribute to filling this knowledge gap by presenting a new data set of mining extents derived by visual interpretation of satellite images. Our data set covers more than six thousand mining sites distributed across the entire globe. These mining sites have reported mineral extraction or activities between the years 2000 and 2017, according to the SNL Metals and Mining database^19^. Within these regions, we delineated the mining areas (i.e., drew polygons) by visual interpretation of several satellite data sources, including Google Satellite, Microsoft Bing Imagery and Sentinel-2 cloudless^33^. As a result, we derived a set of 21,060 polygons globally, covering a total area of 57,277 *km*^2^. The overall accuracy, calculated from 1,000 stratified random points is 88.4% (for details see the section on Technical Validation).

This novel data set can help improving environmental impact assessments of the global mining sector, for example, regarding mining-induced deforestation or fragmentation and degradation of ecosystems. It can also serve as a benchmark for further monitoring the temporal evolution of mining sites around the world and as training and validation data to support automated classification of mines using satellite images.

## Methods

We produced the global-scale data set on mining areas by visual interpretation of satellite images. This remote sensing technique is precise but also costly and time-intensive. To make the visual interpretation viable on a global scale, we defined regions of interest (ROI) based on the SNL Metals and Mining database^19^. This was important to reduce the time spent inspecting the satellite images and delineating the mining extents. Automated post-processing was also applied to check and correct possible invalid polygon geometries^34^, for instance polygons with self-intersections.

### Region of interest

We defined our ROI as a buffer around the geographical coordinates (georeferenced points) of active mines reported in the SNL Metals and Mining database^19^. The SNL database provides production information on more than 35,000 mines across the globe. Among many other variables, SNL reports the approximate geographic coordinates of the extraction sites, from which we selected all mines reporting activity (i.e., actual production or active status) at any time between the years 2000 and 2017. This subset added up to 6,021 mining locations extracting 76 different commodities, with a focus on coal, metal ores and industrial minerals. Note that many mines, particularly regarding metal ore extraction, report more than one commodity in the SNL database (see full list in Table 1).

**Table 1:** List of commodities from active mines reported in the SNL database^19^.

Commodity name (Number of mines reporting the commodity)		
Coal (3119)	Zircon (30)	Yttrium (4)
Gold (1500)	Vanadium (29)	Barite (3)
Silver (1002)	Heavy Mineral Sands (28)	Beryllium (3)
Copper (837)	Lanthanides (28)	Caesium (3)
Zinc (524)	Rutile (28)	Ferrotungsten (3)
Iron Ore (474)	Iridium (27)	Garnet (3)
Lead (450)	Ruthenium (25)	Iron Sand (3)
Nickel (197)	Titanium (24)	Mercury (3)
Cobalt (160)	Tantalum (21)	Osmium (3)
Molybdenum (131)	Niobium (16)	Rubidium (3)
Diamonds (129)	Leucoxene (13)	Scandium (3)
U3O8 (109)	Spodumene (13)	Selenium (3)
Platinum (90)	Magnesium (12)	Gallium (2)
Bauxite (85)	Arsenic (9)	Germanium (2)
Chromite (85)	Bismuth (9)	Kaolin (2)
Palladium (82)	Cadmium (9)	Lanthanum (2)
Manganese (78)	Rhenium (9)	Limestone (2)
Magnetite (62)	Chromium (7)	Beryl (1)
Phosphate (54)	Graphite (6)	Hematite (1)
Rhodium (53)	Tellurium (6)	Potassium Oxide (1)
Tungsten (51)	Ferrochrome (5)	Potassium Sulfate (1)
Tin (44)	Ferronickel (5)	Rare Earth Elements (1)
Potash (41)	Ferrovanadium (5)	Silica (1)
Antimony (38)	Cerium (4)	Strontium (1)
Ilmenite (34)	Indium (4)	
Lithium (30)	Thorium (4)	

The buffer around the selected SNL mines was necessary to increase the efficiency and systematize the interpretation of the satellite images. The radius of the buffer should be as small as possible and cover all mining ground features, including open cuts, tailings dams, waste rock piles, water ponds, and processing infrastructure. Besides, the size of the buffer should consider that the geographical coordinates reported in the SNL database can differ between 1 *km* and 3 *km* from the mines identified in satellite images^10,14^.

After inspecting a random selection of mines we found that a 10 *km* radius was adequate for our propose, i.e., covering all ground features related to the mines while minimizing the time spent on the visual interpretation of the images. The 10 *km* buffer was sufficient to cover most of the mining complexes spreading over several kilometers, including the largest mines in the world, which have an open cut extending over 4 *km* diameter.

### Delineation of mines

The polygons were delineated by two trained experts using an open-source web application^35^ developed for this specific purpose. The web interface systematically displays buffers and markers with information about the mines. As background, the app offers three options of satellite layers: Google Satellite, Microsoft Bing Imagery, and Sentinel-2 cloudless^33^. Google Satellite and Microsoft Bing provide images with a spatial resolution finer than 5 *m* for many regions of the world. These images allow identifying ground features related to mines with high confidence^9^. However, these data sources do not cover the whole globe with the same spatial resolution and contain out-of-date images for some regions^36^. To fill this gap, we used the Sentinel-2 cloudless data product with a 10 *m* spatial resolution provided by EOX^33^. The Sentinel-2 cloudless provides a mosaic built from Sentinel-2 images taken during the years 2017 and 2018. Combining these data layers, the experts identified and delineated the ground features related to mining.

All three satellite data sources were visually inspected before delineating the polygons. The majority of the inspected locations had at least two sources of clear images (e.g., no cloud cover) and sufficient spatial resolution to identify mining features. Only very few locations lacked images with sufficient quality to draw the polygons, for example, due to cloud cover or low spatial resolution.

We used the source showing the largest mining extent for the delineation of the areas. This premise was taken because the largest extent of a mine is usually stable for several years as a long lifespan is intended due to economic reasons. Besides, mining areas generally increase and could only reduce through ecological restoration, which can take a long time^37^. These conjectures do not ensure the temporal consistency of all delineated extents but helped to capture the largest and most up-to-date extent of the mines according to the available satellite images within our ROI.

In some cases, the mining polygons can also extend beyond the ROI. Mining features intersecting the buffer borders were delineated to account for their full extent, even if they extend beyond the buffer limits. Moreover, the mining polygons can contain isolated patches with forest or other land covers, which do not necessarily represent any mining feature on the ground. These patches were included because we aim at accounting for the total area used by mining, including isolated spare areas that most probably cannot have other uses. The delineated polygons do not distinguish the different ground features within the mines, i.e., each polygon can cover several mining features (open cuts, tailings dams, waste rock dumps, etc). As a final product from the delineation we obtained a set of polygons covering the total land used by mining within the ROI.

### Geoprocessing of data records

We applied geospatial and geometric operations to check and correct the raw data collection. This geoprocessing was performed to avoid double counting of mining areas, correct invalid geometries, and add attributes (variables) to the polygons. To avoid double-counting, we dissolved polygons that possibly overlapped or shared a common boundary, i.e., we merged them to form a single polygon. After that, we removed *sliver polygons* (unwanted small polygons) and invalid polygon geometries, producing a consistent set of polygons.

From this set of preprocessed polygons, we calculated the area of each feature and added information on the country where each polygon is located. We calculated the area in square kilometers by projecting each polygon to its respective Universal Transverse Mercator (UTM) zone. After that, a spatial join query acquired country name and ISO 3166-1 alpha-3 code from country’s administrative units geometries available from EUROSTAT^38^. The final set of polygons thus includes the geometries (polygons) covering the mining areas, their respective areas in square kilometers, country name, and ISO 3166-1 alpha-3 code of the corresponding country.

From the mining polygons we derived global grid data sets with the mining area at 30 *arcsecond*, 5 *arcminute* and 30 *arcminute* spatial resolution (approximately 1 × 1 *km*, 10 × 10 *km* and 50 × 50 *km* at the equator). This is useful because many modeling applications require standardized grid data^39^. The 30 *arcsecond* grid was derived from the percentage of area of the geometric intersection between each cell and the geometries of the mining polygons. These percentages were rounded to zero decimal digits to reduce the size of the data set. Therefore, the percentage of the cell covered by mine should be greater than 0.5% to be considered, i.e., approximately 0.5 *ha* at the equator. To obtain the gridded mining area, we estimated the area of each cell in square kilometers and multiplied with the percentage of mining cover per cell, resulting in a 30 *arcsecond* global grid indicating the mining area within each cell. The 5 *arcminute* and 30 *arcminute* grid resolutions were downsampled form the 30 *arcsecond* grid. All scripts used in the geoprocessing of data records are available with our open-source web application tool^35^.

## Data Records

Our data records provide spatially explicit information on the direct land use of mining activities. The main data set consists of 21,060 mining polygons covering the extents of mining sites worldwide^40^. Grid data derived from the polygons is available at 30 *arcsecond*, 5 *arcminute*, and 30 *arcminute* spatial resolution, providing a ready-to-use data set for modeling purposes with the mining area in square kilometers per grid cell. All data records are available for download from PANGAEA (Data Publisher for Earth & Environmental Science) at 10.1594/PANGAEA.910894 and for visualization at https://www.fineprint.global/viewer.

### Mining polygons

**Fig. 1:**
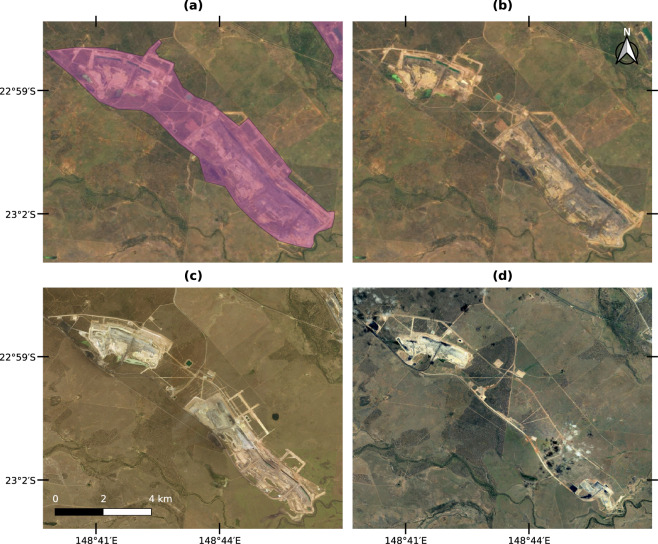
An example polygon delineated over a coal mine in Mackenzie River, Queensland, Australia. (**a**) Shows the delineated polygon in purple and (**b**) shows the Sentinel-2 cloudless mosaic composed by images from the year 2018^33^ used to delineate the mining extent. (**c**) Shows a Microsoft Bing image from July 2011 and (**d**) a Google Satellite image from December 2007.

Figure 1 illustrates how the satellite images were used to delineate the mining extent. In this example, the area is used for coal mining in Mackenzie River, Queensland, Australia. The polygon in Fig. 1a was derived from the Sentinel-2 cloudless mosaic (Fig. 1b), which shows the largest extent of the mine among all three images sources. The Sentinel-2 cloudless mosaic is composed by images from the years 2017 and 2018^33^ while Microsoft Bing (Fig. 1c) and Google Satellite (Fig. 1d) only offered out-of-date images for that location, respectively taken in July 2011 and December 2007. Nevertheless, all three data sources contributed to providing pieces of evidence of mining in the mapped area.

**Fig. 2:**
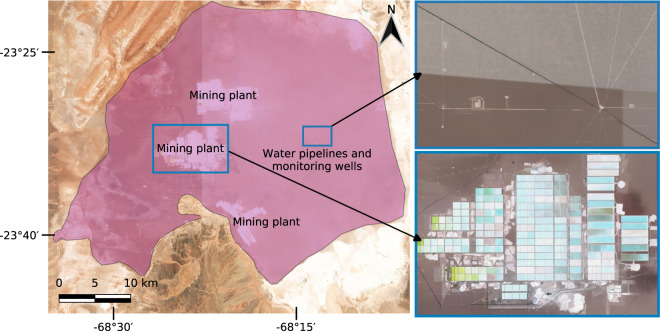
Mine on the *Salar de Atacama* salt flat, Chile. The purple polygon on the left side was derived from the Sentinel-2 images shown in the background. The polygon covers all infrastructure spread over the salt flat, including water pipelines, wells, and the actual mining plants. The zoom boxes on the right side show Google Satellite images with a detailed view of water pipelines and wells over the salt flat as well as one of the mining plants.

The delineated polygons cover all infrastructure and land cover types directly related to mining activities. This can produce large polygons, such as in the case of the *Salar de Atacama*, Chile. In that area, we delineated a polygon of approximately 1,354 *km*^2^, covering almost the whole nucleus of the salt flat, which extends over 1,360 *km*^2^ and is used as a source to extract lithium, boron, potassium, iodine, sodium chloride, and bischofite^41^. Figure 2 shows the delineated polygon extent and a detailed view of one of the mining plants. Some pipelines and wells are more than 10 *km* away from the core infrastructure of the mine. We decided to map the whole area because the mining plants, in fact, have brine pumping and monitoring wells spreading over the entire salt flat far beyond the actual evaporation ponds^41^. Alternative assumptions mapping only the evaporation ponds estimated an area of only 80.53 *km*^2^ in 2017^42^. However, it is important to note that the case of *Salar de Atacama* was rather isolated; in most cases, no features such as pipelines and wells outside the main mining sites could be identified from the available satellite images.

**Fig. 3:**
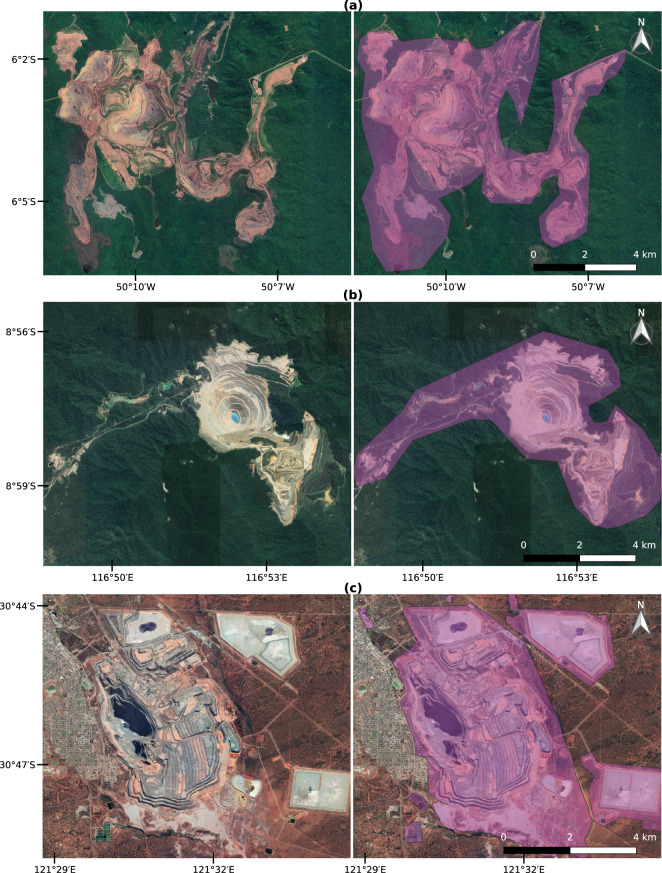
Examples of mapped mining polygons with Google Satellite images background. (**a**) Carajás iron ore mine in Brazil, (**b**) Batu Hijau copper-gold mine in Indonesia, and (**c**) Super Pit gold mine in Australia.

In many cases, mines are located following the structure of mineral deposits, making it easy to map them from satellite images. We selected three mines to illustrate these large-scale concentrated activities (Fig. 3). The first example (Fig. 3a) shows the main open cut of the Carajás iron ore mine complex in the Brazilian Amazon, which is among the world’s largest iron ore mining operations^43^. Figure 3b shows the Batu Hijau copper-gold mine. Despite its large open cut, this mine does not use much area for unused material, as its tailings disposal takes place in the ocean^44^. The third example is the Super Pit gold mine in Australia, Fig. 3c. This mine is located in one of the largest gold producing regions in the world. In the case of these large mines, coordinates reported in the SNL database were accurate.

**Fig. 4:**
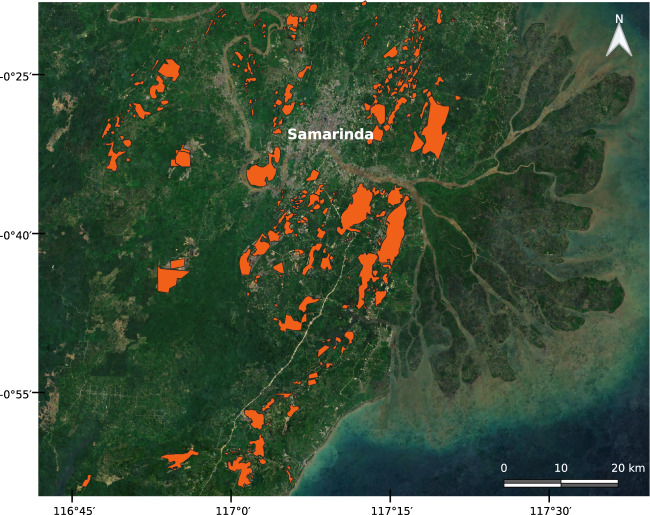
Coal mining polygons in East Kalimantan, Indonesia, overplayed with the Sentinel-2 Cloudless images form the year 2019 provided by EOX^33^.

Contrasting to the above examples, in other regions the reported coordinates were of lower accuracy. Figure 4, for example, shows a large area with widely spread coal mining activities in East Kalimantan, Indonesia. The SNL database reports some mining locations in this region, however, they do not always spatially intersect the mining areas mapped from the satellite images. In these cases the predefined ROI (10 *km* buffer around the coordinates) was crucial to systematically map the extents of the mines.

### Overview of global mapped mining area

**Fig. 5:**
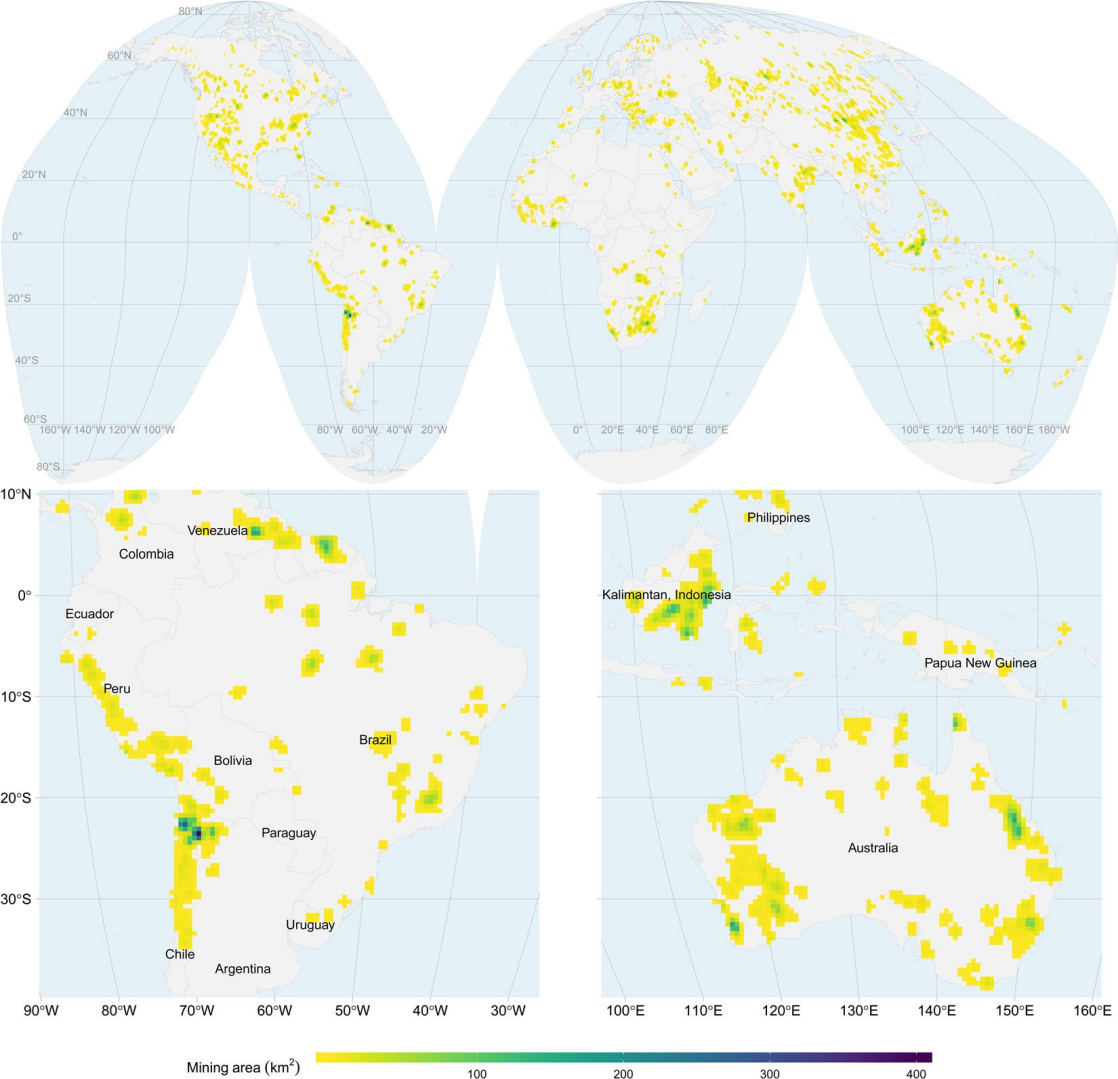
Mining area aggregated to 50 *km* grid cells projected to Interrupted Goode Homolosine. The map at the top shows the global distribution of the mapped mining area. The maps at the bottom are zoomed to South America, Australia, and parts of South-East Asia.

Figure 5 shows an overview of the geographical distribution of our mapped mining area across the globe. The map in the figure is projected to equal area Interrupted Goode Homolosine and resampled to a 50 × 50 *km* grid to facilitate visualization. From this figure we can see concentrations of mining areas in many regions, for example, in northern Chile mainly due to copper extraction and northeastern Australia and East Kalimantan in Indonesia because of coal mining.

**Fig. 6:**
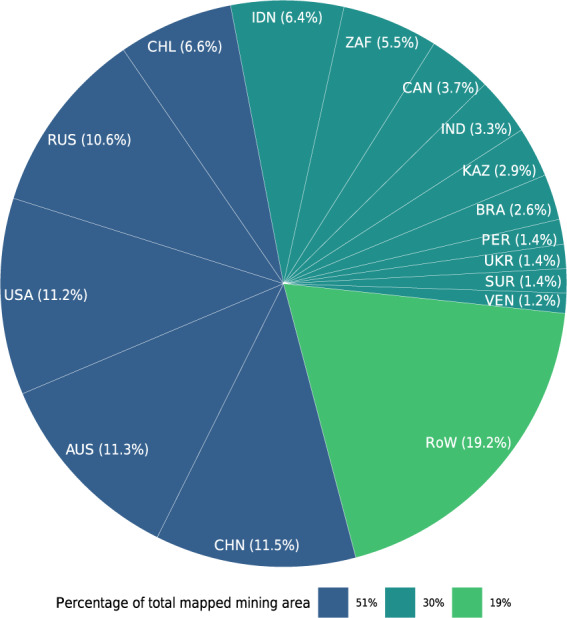
Percentage of mining area mapped per country. The colors represent groups of countries covering 51%, 30%, and 19% of the mapped area.

A summary of our data aggregated by country shows that 51% of the mapped mining area is concentrated in only five countries: China, Australia, the United States, Russia, and Chile. Another ten countries account for 30%, and the remaining countries add up to 19% of the total mapped mining area (Fig. 6). These results show that mining areas are highly concentrated in only a few countries. However, it is worth mentioning that our polygons could be biased by the activities reported in the SNL database and could mask countries and commodities that are poorly reported. For instance, SNL data underestimates the quantities extracted in China for most metals and minerals compared to national accounts according to UNEP’s Global Material Flows Database^2^. For most African countries, however, SNL extraction of metals compares well to the national aggregates. One of the few exceptions is gold from the DR Congo, where SNL data sums up to less than 6 *mt* in the year 2017, while UNEP reports more than 10 *mt* of gold ore extraction.

Countries have different profiles regarding the spatial distribution of the mines. For example, China and Australia have similar figures on the mapped mining area, 6,567 *km*^2^, and 6,470 *km*^2^. However, they vary with respect to the number of identified polygons, 5,557 and 1,797, respectively. This discrepancy in the number of mining locations can be related to the high importance of the small-scale mining industry in China^45,46^, while Australia is characterized by fewer, large-scale mines^19^.

**Fig. 7:**
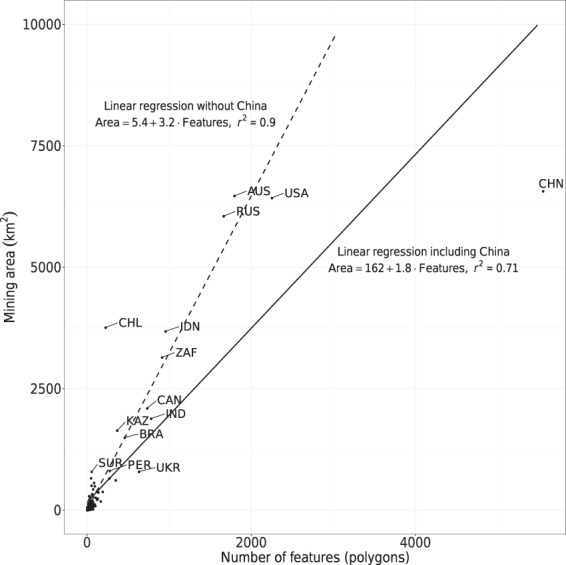
Relationship between the mapped mining area and the number of features (polygons) on a country level. The solid line summarizes the relationship between area and number of features for the complete data set, the dashed line excludes China.

Figure 7 displays the relationship between the mapped area and the number of polygons on a country level. Most of the variation in mining area can be explained by a linear relationship to the number of polygons. Excluding China from the data set, a simple linear regression model reaches *r*^2^ = 0.90 (dashed line in Fig. 7). However, *r*^2^ drops to = 0.71 for the full data set including China (solid line in Fig. 7). A complete summary of the mining area mapped per county is shown in Table 2 and available from download with our data records^40^.

**Table 2:** Mining area in *km*^2^ and number of polygons (n) mapped per country.

Country	*km*^2^	n	Country	*km*^2^	n	Country	*km*^2^	n
CHN	6,567.15	5,557	GIN	146.56	67	ITA	13.70	43
AUS	6,470.29	1,797	BOL	142.04	36	TJK	13.42	12
USA	6,427.74	2,252	THA	130.72	13	PAK	12.94	17
RUS	6,053.14	1,665	SRB	130.10	41	OMN	12.85	46
CHL	3,759.66	227	GBR	120.09	95	FRA	12.83	7
IDN	3,681.40	957	SWE	114.00	46	MDG	12.33	9
ZAF	3,145.97	915	FIN	104.24	77	LBR	11.65	18
CAN	2,099.07	734	TZA	98.57	19	LSO	11.65	8
IND	1,884.96	781	NZL	89.74	102	PAN	11.43	7
KAZ	1,640.33	368	ESP	88.77	69	MNE	10.88	13
BRA	1,493.36	459	MRT	85.75	25	ERI	10.15	4
PER	809.75	277	BFA	81.82	30	SVK	10.07	28
UKR	792.49	634	SLE	76.39	63	GTM	9.72	13
SUR	791.42	57	MOZ	75.57	21	NIC	9.48	9
VEN	658.93	50	CUB	71.10	25	AUT	9.36	7
GHA	657.50	273	MAR	68.33	26	URY	9.20	4
MEX	617.60	350	MYS	63.38	53	IRQ	8.84	4
ARG	564.58	86	PNG	62.98	17	TUN	8.54	4
COL	507.70	56	HUN	62.20	75	PRT	8.30	13
DEU	496.04	96	ESH	61.73	1	ETH	7.22	5
ZMB	432.56	75	KGZ	61.53	23	ALB	7.20	21
MNG	384.15	119	EGY	49.21	10	HND	7.15	7
TUR	378.74	191	SAU	47.65	50	AZE	7.11	12
COD	367.38	139	NER	46.63	10	IRL	6.76	8
NAM	331.05	63	GAB	46.07	7	ECU	6.61	20
IRN	321.34	60	LAO	45.66	10	KEN	5.24	5
UZB	290.02	25	ISR	44.17	9	SLB	5.05	1
JOR	263.40	45	MMR	40.45	9	JPN	4.84	12
POL	256.69	105	CIV	37.21	9	CYP	4.24	2
NCL	238.03	124	ARM	30.07	36	ARE	4.21	6
GUY	237.14	132	NOR	28.68	16	MWI	3.89	4
BWA	225.15	29	DOM	28.62	15	SVN	1.75	4
PHL	217.94	124	BIH	24.90	7	BGD	1.27	2
GRC	194.99	56	SEN	23.93	7	FJI	1.24	7
BGR	188.19	40	JAM	22.37	35	RWA	1.24	7
ZWE	182.41	167	PRK	20.94	17	CRI	1.04	2
AGO	179.22	75	DZA	19.89	75	SJM	1.04	3
VNM	160.37	70	MKD	18.17	12	UGA	0.22	2
MLI	157.03	30	KOR	17.67	18	GNB	0.06	2
CZE	156.30	48	SDN	14.72	15			
ROU	154.87	56	GEO	14.37	7			

Total Area: 57,277.73 *km*^2^; Total number of features: 21,060.

Our mining data set accounts for all land cover types related to mining that could be identified from the satellite images. However, it does not distinguish the different features within the polygons. For example, we could not separate mining from quarry, because this would require additional information other than the satellite images. Although our data set does not cover all existing mines, to date, it is the most comprehensive database on mining extents openly available. The data set can help filling existing gaps for spatially explicit mineral extraction assessments on a global scale. It opens up opportunities to improve environmental pressure and impact indicators of the mining sector and can support the development of automated systems to monitor mining sites worldwide.

## Technical Validation

The mapped mining extents presented in this work can be subject to many sources of error, ranging from experts’ interpretation to the temporal availability and precision of the satellite images. The precision of the delineated mining borders can vary according to the satellite data source and the location. In general, the satellite sources used in this work provide sufficient spatial resolution and georeferencing accuracy to map mining areas^9^. Images available from Google Earth, for instance, have an overall positional root mean squared error (RMSE) of 39.7 *m* related to the reality on the ground^47^. Sentinel-2, on the other hand, has a RMSE below its pixel size (10 × 10 *m*)^48^. These errors are acceptable for global scale environmental assessments.

The visual interpretation of satellite images depends on the previous knowledge of the perceiving person. The ground features related to mining are not always easy to identify in the satellite images and can be subject to the judgment of the person that delineates a particular mine. For that reason, we obtained a second independent classification for a set of random points. We drew a set of 1,000 random points stratified^49^ between the area mapped as mine and those not mapped as mine (no-mine) within the region of interest (10 *km* buffer from the geographical coordinates). These validation points were inspected independently by experts that did not participate in the delineation of the mines. They classified these validation points as mine or no-mine based on the three satellite data sources without information whether or not the points were originally mapped as part of a mining areas. The validation points are also part of our data records^40^.

The overall agreement between the mapped areas and the validation points was 88.4%. Assuming that the validation points consist of a reference data set, we derived User’s (commission errors) and Producer’s (omission errors) accuracy (see Table 3). The User’s accuracy tells how well the classes in the map represent the reality on the ground; the Producer’s accuracy points how well a class has been mapped^50^. In our case the mapped mining areas have 97.5% User’s accuracy and 78.8% Producer’s accuracy, meaning that the mapped areas are highly reliable (less than 3% was incorrectly mapped as mining), but we missed some mining areas (the omission of mines was around 21.2%). The omission of mines also reflects a lower User’s accuracy of the no-mine class (82.2%).

**Table 3:** Error matrix and accuracy statistics derived from 1,000 random points equally allocated between the mapped classes mine and no-mine.

Mapped	Reference			User’s acc. (%)
	Mine	No-mine	Total	
Mine	394	106	500	97.5
No-mine	10	490	500	82.2
Total	404	596	1000	
Producer’s acc. (%)	78.8	98.0		

Overall acc.: 88.4%; Kappa: 0.77; F1 Score: 0.87.

**Fig. 8:**
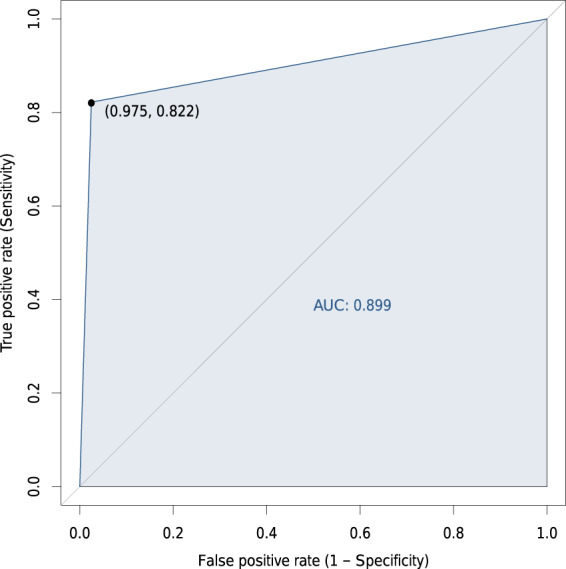
Receiver Operating Characteristic (ROC) derived from 1,000 random points equally allocated between the mapped classes mine and no-mine. The point in the ROC curve shows the performance of our binary (mine/non-mine) classification and the shade shows the area under the ROC curve (AUC).

An alternative way to visualize the accuracy of our data set is the Receiver Operating Characteristic (ROC probability curve). The graph in Fig. 8 displays the classification performance in terms of *true positive* and *false positive*. A discrete classifier (mine/no-mine) produces a point in the ROC curve. For our classification, the point is near the upper-left corner of the ROC curve, meaning that the classification performs well (a perfect classifier would reach the point 0, 1). Besides, the area under the curve (AUC) in Fig. 8 shows that our classification has 89.9% probability of correctly distinguishing between mine and no-mine.

Looking at the spatial distribution of the validation points, we found that half of the points with disagreement (i.e., 58 points) are located less than 50 *m* from the borders of the delineated polygons. On the other hand, of the points with an agreement (i.e., 884 points) only 16% are located closer than 50 *m* to the polygons’ borders. This shows that higher uncertainty lies on the borders of the delineated extents as it can be expected due to the use of several satellite data sources with different precision. These results also indicate that we have high confidence in the existence of mines within the mapped polygons.

## Usage Notes

The global mining data set described here is available from PANGAEA under the license Creative Commons Attribution-ShareAlike 4.0 International (CC-BY-SA). The data records include the mining polygons, validation points, mining area grid, and a summary of the mining area per country.

1. The mining polygons and validation points are encoded in *GeoPackage* geographic data structures^51^, such as:

(a) the *mining_polygons* layer has five attributes:

- ISO3_CODE: A string with the country’s ISO 3166-1 alpha-3 code
- COUNTRY_NAME: A string with the country name in English
- AREA: A number with the area of the feature in square kilometers
- geom: A polygon geometry in geographical coordinates WGS84
- fid: An integer with feature ID

(b) the *validation_points* layer has four attributes:

- MAPPED: A string with the class derived from the mining polygons (“mine” or “no-mine”)
- REFERENCE: A string with the validation class (“mine” or “no-mine”)
- geom: A point geometry in geographical coordinates WGS84
- fid: An integer with feature ID

2. The mining grids include a single layer (one band raster) encoded in Geographic Tagged Image File Format (GeoTIFF)^52^. Each grid cell over land has a float number (data type *Float32*) greater than or equal to zero representing the mining area in square kilometers; grid cells over water have no-data values. The grid is available in three spatial resolutions, 30 *arcsecond*, 5 *arcminute*, and 30 *arcminute*, extending from the longitude −180 to 180 degrees and from the latitude −90 to 90 degrees in the geographical reference system WGS84.

3. The summary of the mapped mining area per country derived from the mining polygons is available in Comma-separated values (CSV)^53^ format, including four attributes:

- COUNTRY_NAME: A string with the country name in English
- ISO3_CODE: A string with the country ISO3 code
- AREA: A number with the area of the feature in square kilometers
- N_FEATURES: An integer with the number of features per country

Our spatially explicit data records can be combined with other geographical data to perform further statistical analysis, for example, to test spatially stratified heterogeneity^54^ and non-stationarity of variables^55,56^. For that, users can open the data records using software that support Geographic Information System (GIS), including, QGIS^57^, R^58^, and Python^59^. Besides, we also provide a tool for visual analysis of the geographical data records at www.fineprint.global/viewer and a Web Map Service (WMS)^60^ accessible from www.fineprint.global/geoserver/wms.

## Data Availability

All the code and geoprocessing scripts used to produce the results of this paper are distributed under the GNU General Public License v3.0 (GPL-v3)^61^ from the repository www.github.com/fineprint-global/app-mining-area-polygonization^35^. The processing scripts were written in R^58^, Python^59^, and GDAL (Geospatial Data Abstraction Library^62^). The web application to delineate the polygons was written in R Shiny^63^ using a PostgreSQL^64^ database with PostGIS^65^ extension for storage. The full app setup uses Docker^65^ containers to facilitate management, portability, and reproducibility. The web application supports the delineation of areas from the satellite images layers. It systematically displays the regions of interest (e.g., buffer around the mines) and several background options of satellite images, which the users can take into account to draw and edit polygons. Note that mining coordinates are not part of the web application and must be fed into the database by the user. To learn more about the application setup see www.github.com/fineprint-global/app-mining-area-polygonization. The current version of app provides image layers from Sentinel-2 Cloudless^33^, Google Satellite, and Microsoft Bing Imagery. Further sources of satellite images can be added to the application via WMS.
